# CRISPR-Cas9 HDR system enhances AQP1 gene expression

**DOI:** 10.18632/oncotarget.22901

**Published:** 2017-12-04

**Authors:** Zhimin Wang, Yaohe Wang, Songling Wang, Li-Rong Zhang, Na Zhang, Zhenguo Cheng, Qingshi Liu, Kelly J. Shields, Baoli Hu, Michael J. Passineau

**Affiliations:** ^1^ Sino-British Research Centre for Molecular Oncology, National Centre for International Research in Cell and Gene Therapy, School of Basic Medical Sciences, Academy of Medical Sciences, Zhengzhou University, Zhengzhou 450052, China; ^2^ Centre for Molecular Oncology, Barts Cancer Institute, Queen Mary University of London, London EC1M 6BQ, UK; ^3^ Salivary Gland Disease Center and Molecular Laboratory for Gene Therapy and Tooth Regeneration, School of Stomatology, Capital Medical University, Beijing 100069, China; ^4^ Department of Pharmacology, School of Basic Medical Sciences, Zhengzhou University, Zhengzhou 450052, China; ^5^ Department of Medicine, Lupus Center of Excellence-Autoimmunity Institute, Allegheny Health Network, Pittsburgh, PA 15212, USA; ^6^ Division of Neurosurgery, Children’s Hospital of Pittsburgh of UPMC, Pittsburgh, PA 15224 USA; ^7^ Department of Neurological Surgery, University of Pittsburgh School of Medicine, Pittsburgh, PA 15261, USA; ^8^ Gene Therapy Program, Cardiovascular Institute, Allegheny Health Network, Pittsburgh, PA 15212, USA

**Keywords:** replacement of promoter, gene editing, aquaporin 1, salivary gland dysfunction

## Abstract

Ionizing radiation (IR) isthe primarytherapeutic tool to treat patients with cancerous lesions located in the head and neck. In many patients, IR results in irreversible and severe salivary gland dysfunction or xerostomia. Currently there are no effective treatment options to reduce the effects of xerostomia. More recently, salivary gland gene therapy utilizing the water-specific protein aquaporin 1 (AQP1) has been of great interest to potentially correct salivary dysfunction. In this study, we used CRISPR-Cas9 gene editing along with the endogenous promoter of AQP1 within theHEK293 and MDCK cell lines. The successful integration of the cytomegalovirus (CMV) promoterresultedin a significant increase of AQP1 gene transcription and translation. Additionalfunctional experiments involvingthe MDCK cell line confirmedthat over-expressed AQP1increasedtransmembrane fluid flux indicative of increased intracellular fluid flux. The off-target effect of designed guided RNA sequence was analyzed and demonstrateda high specificity for the Cas9 cleavage. Considering the development of new methods for robust DNA knock-in, our results suggest that endogenous promoter replacement may be a potential treatment forsalivary gland dysfunction.

## INTRODUCTION

Each year in the United States there are an estimated 40,000 cases of head and neck cancer. For the majority of these patients, ionizing radiation is a fundamental element of therapy; however, the patientsoften suffer from the impairment of normal salivary gland function [1, 2, 3]. The salivary glands play important roles in oral health aiding in food digestion and protecting oral mucosa. Manycancer patients experience a dramatic decrease in the quality of life. Our research targets the restoration of normal salivary gland function to improve the quality of life for these cancer survivors [3, 4].

Prescription medications and moisturizers are available to reduce side-effects of the ionizing radiation on salivary gland function. Current areas of research to restore normal salivary gland function include1) regeneration,2) the use of ultrasound-assisted gene therapy (UAGT) targeting aquaporin (AQP1), and 3) epigenetic modifications. [5, 6, 7, 8, 9, 10, 11].

Recently, a new programmable RNA-guided nuclease has been discovered and has the potential to be arevolutionary toolbox for both single cells andorganisms. Originally derived from the bacterial adaptive immune systems, this methodutilizes clustered regularly interspacedshort palindromic repeats (CRISPR)-associate protein (Cas9), which opens a nearly infinite number of genome engineering opportunities [12]. In brief, the Cas9 endonuclease is led by a guide RNA (gRNA) to cleave and induce double strand breaks (DSBs) at homologous sites. In subsequent genome editing, the DSBs are fixed by two different repair mechanisms, including the predominant error-prone non-homologous end joining (NHEJ) and the less-efficient templated homologous-directed repair (HDR) [13, 14]. Due to its broad application, CRISR-Cas9 technology has been used to evaluate gene function in prokaryotes to eukaryotes with the potential to influence treatment options for gene therapy in humans [15, 16, 17].

In this study, we aim to enhance the expression of endogenous AQP1using the novel CRISPR-Cas9 system bydesigning agRNA sequence and cytomegalovirus (CMV) containing homology directed repair (HDR) template. We use the Human Embryonic Kidney-293 (HEK293) cell linefor proof-of-principle and to evaluate off-target effects (OT) and the Madin-Darby Canine Kidney Epithelial (MDCK) cell line to conduct a functional fluid flux assay to verify the altered cells demonstrate a change intheir permeability mimicking a restored salivary gland state.

## RESULTS

### CRISPR-Cas9 system identified gRNA1 with highest cleavage efficiency in HEK293 cells

**Figure 1:**
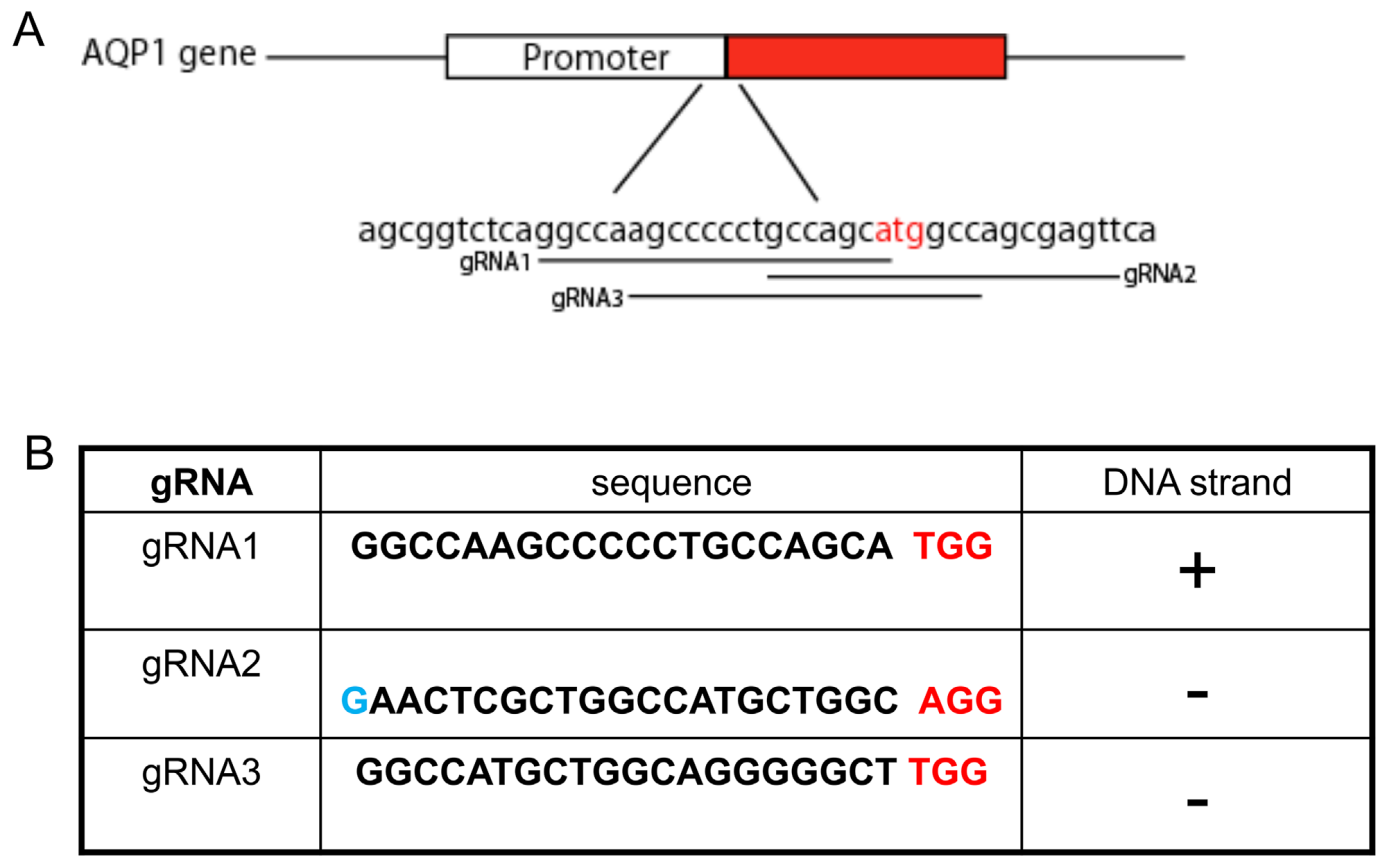
Guided RNAs design of human AQP1gene **(A)** The location of three gRNAs of human AQP1 gene in chromosome 7 was indicated, and the start codon ATG is highlighted in red. **(B)** The sequence of gRNAs and the chromosome targeting orientation are demonstrated. The human U6 promoter (pol III promoter) prefers to use the G nucleotide as the start transcription site to attain higher transcription efficiency [28]. To ensure this, an extra G nucleotide (highlighted in blue) was added to the start of target sequence for the gRNA2. The PAM sequence is underlined.

**Figure 2:**
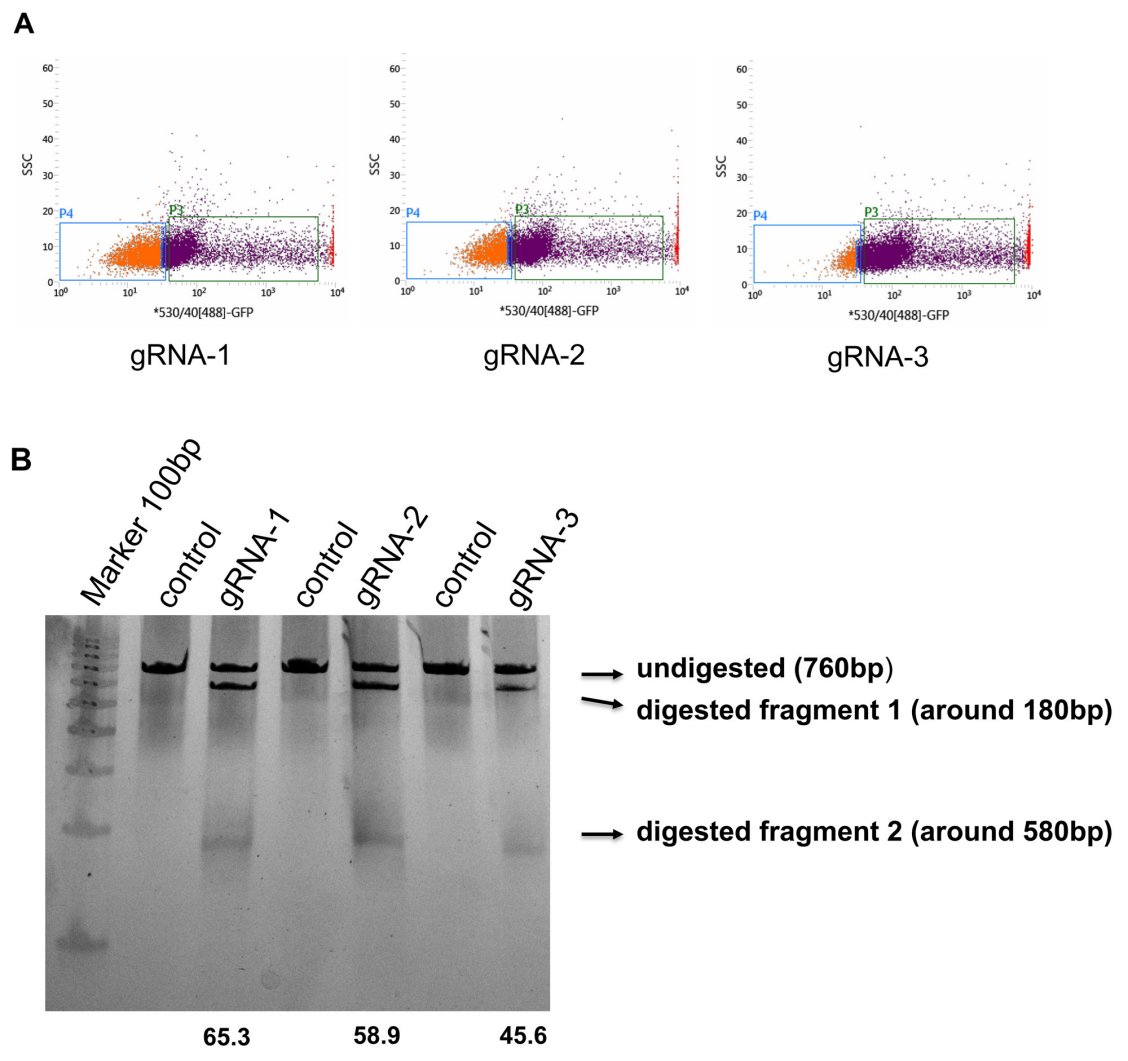
Assessing the editing efficiency of the human AQP1 gRNAs **(A)** PX458 vectors containing human AQP1 gRNAs were transfected into HEK293cells and GFP positive cells were sorted by FACS. **(B)** The sorted cells and non-transfected cells (negative control) were collected, and DNA genomes were isolated. The fragments crossing the separate gRNA sequenceswere amplified and further digested with T7 endonuclease. Note the cleavage efficiencieswere calculated and shown at the bottom of their respective gRNA-lane. The location and size of potential cleavage fragments are indicated using an arrow to the right of image.

First, we sought to find three potentialgRNA sequences to induce DSBs within the vicinity of AQP1transcription start site(TSS) using the CRISPR design tool (Figure 1). Vectors (PX458 with green fluorescent protein (GFP)) expressing these gRNAs were transfected into HEK293 cells. Using flow cytometry techniques, we found the sorting efficiency to be similar for the three separate gRNA sequences (Figure 2A).

We used the T7 Endonuclease I assay gel to visualize the bands of the respective digestive fragmentsand determine that all three gRNAs generated DSBs (Figure 2B). Additionally, we quantified the cleavage efficiency using the same gel: gRNA1: 65.4%, gRNA2:58.9%, gRNA3:45.6% (Figure 2B). We chose the gRNA1 with thehighest cleavage efficiency for further experiments.

### gRNA1 was specific for AQP1 gene cleavage: no off-target (OT) cleavage in HEK293 cells

**Figure 3:**
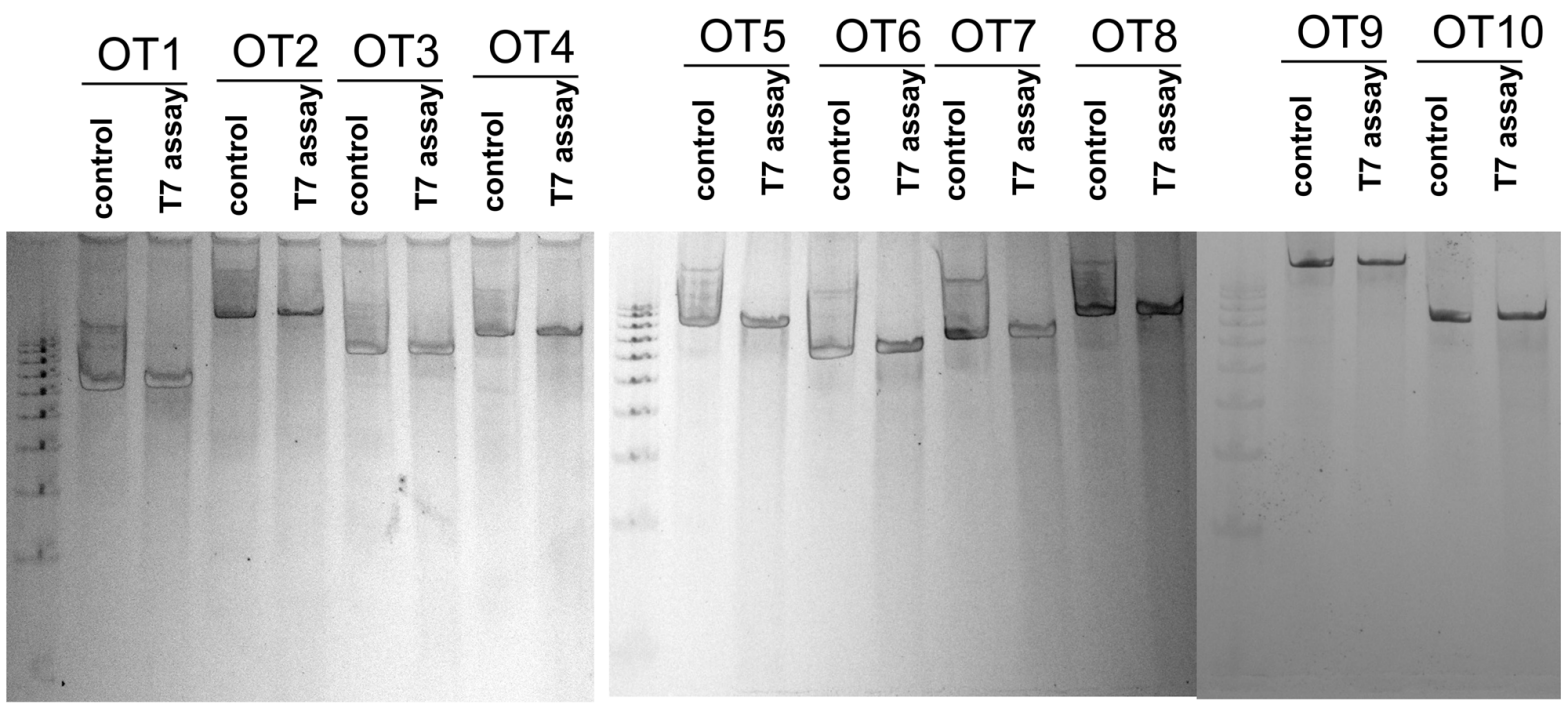
Ten off-target (OT) sequences for gRNA1 The HEK293 cells were transfected with or without gRNA1 vectors. The total DNA was isolated;fragment crossing the potential cleavage sequences were amplified and subjected to T7 endonuclease assay. Both controls, DNA without transfection and DNA with PX458-gRNA1 transfection, were digested with T7 endonuclease for each potential off-target sequence. We observed no cleavage bands after the T7 endonuclease treatment suggesting there are no detectable OT cleavage generated by Cas9 guided by the gRNA1 sequence.

A major concern of using CRISPR is thepotential OTeffect. There are four gene sequences within the coding region and six gene sequences within the non-coding region of the loci (Table 1). To further validate the OT effect of the chosen sequences, pair primers were designed targeting respective genome regions. We observed no cleavage bands after the T7 endonuclease treatment suggesting there areno detectable OT cleavage generated by Cas9 guided by the gRNA1 sequence.(Figure 3).

**Table 1:** The 10 highest potential off-target (OT) genome locations forhuman AQP1, along with their PCR running parameters and potential T7 endonuclease cleavage fragments

Number	UCSC gene	Locus	Forward primer	Reverse primer	PCR size	PCR running paratmeters	T7 endonuclease cleavage fragment
OT-1		chr9:+94598064	**5′- TTCACAGCA GTGCCATTTGC**	**5′- ACTCTGTTG TTTAGCGGGGG**	849	55	587+263
OT-2		chr9:-126119854	**5′- TCATGTTTC CCGCACTCACA**	**5′- GCAGTGTAC CCCACCAACTT**	635	55	144+492
OT-3		chr11:+3223881	**5′- GCCATGGGG AGCTGACTATC**	**5′- CACTGTTCA GCCTCGTTGGA**	711	55	271+440
OT-4	NM_207396	chr1:-6271918	**5′- TTCCGACGG ACTTGCTCTTG**	**5′- GTACAGGCC AGGTCCTCTTTC**	886	52	253+634
OT-5	NM_015307	chr15:-29428563	**5′- TGTGTGATC AAGACAGGCCG**	**5′- CTCCTTCGG GCAATGACGAT**	667	55	495+173
OT-6		chr5:-85058281	**5′- CCAGGTTTG AGGCTTTGGGC**	**5′- CCACGATGG TGACCAGGAGC**	1460	58	1320+141
OT-7		chr20:-55740573	**5′- GGCAGCAGC TTGATTGGTGC**	**5′- CTAGGAGCA GGGTGGGCTCT**	870	60	655+216
OT-8	NM_152463	chr17:-48457737	**5′- CCATGCTGG GCCTGTCCTTT**	**5′- GCATGGAGG GTGCTACGCTT**	1110	58	783+318
OT-9		chr20:+13202037	**5′- TCCCTTCCC AGGCTCTACGG**	**5′- CTCTTAGGTG GGGCAGGGGA**	1384	60	763+622
OT-10	NM_001257189	chr7:+44605210	**5′- AGAGCTCAG GCCAGGGCATA**	**5′- AACCCACTG CGCAGCTTCA**	738	58	292+445

### CMV-2A-Neomycin integrates into designed genome locus

We sought to insert the CMV promoter at gRNA1 (highest cleavage efficiency) to enhance the AQP1 expression. Initially, the CMV- (DNA donor fragment) co-transfected with PX458-gRNA1 in HEK293 cells did not produce a significant change in transcription or protein level(data not shown). The failure to enhance the endogenous AQP1 gene expression may have beendue to the relatively low efficiency homologous directed genetic modification.

**Figure 4:**
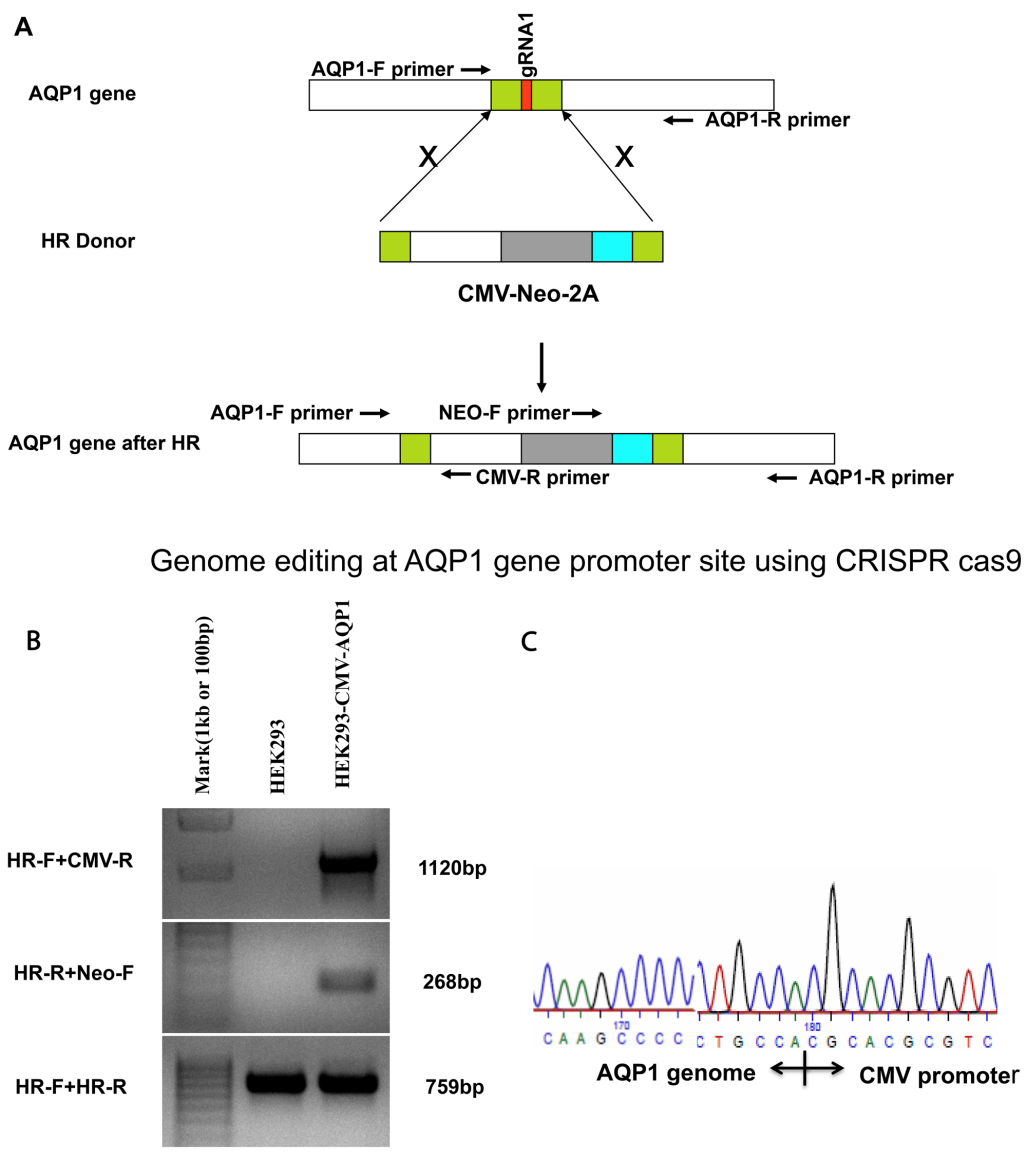
Genome editing at the AQP1 gene promoter site using CRISPR-Cas9 and establishing the integrated stable cell line of HEK293 **(A)** Schematic to introduce the CMV-Neo-2A expression cassette into AQP1 genome locus. Genome editing at the AQP1 gene primers pairs of AQP1 and CMV-R, Neo-F and AQP1-R were used to detect the insertion of expression cassettes, respectively. Primer pair of AQP1-F andAQP1-R was used to confirm the AQP1 gene locus breaks of both alleles. **(B)** Control DNA from HEK293 cells (Lane 2: negative control) and primers shown in (A) were amplified with stable cell DNA (Lane 3). **(C)** The amplified fragment using primer HR-F and CMV-R (Lane 3 in (B)) was isolated by gel extraction and sequenced to confirm the CMV promoter integrated appropriately.

Despite the low efficiency, to continue testing our proof-of-concept, we established a stable cell line to enrich the CMV integrated HEK293 cells. Based on the previously described design, we added a neomycin-2A sequence, between the CMV promoter and RA (Supplementary Figure 2) to drive the neomycin and AQP1 translation while allowing for preferential cleavage at 2A. This strategy ledto a LA-CMV-Neo-2A-RA HR donor fragment under control of the CMV promoter (Figure 4A). We didnot observe a significant difference in cell viability or growth rate between the stable integrated HEK293 cells compared with normal cells after culturing for 2 months, which included30 passages (Supplementary Figures 4 and 6).

**Figure 5:**
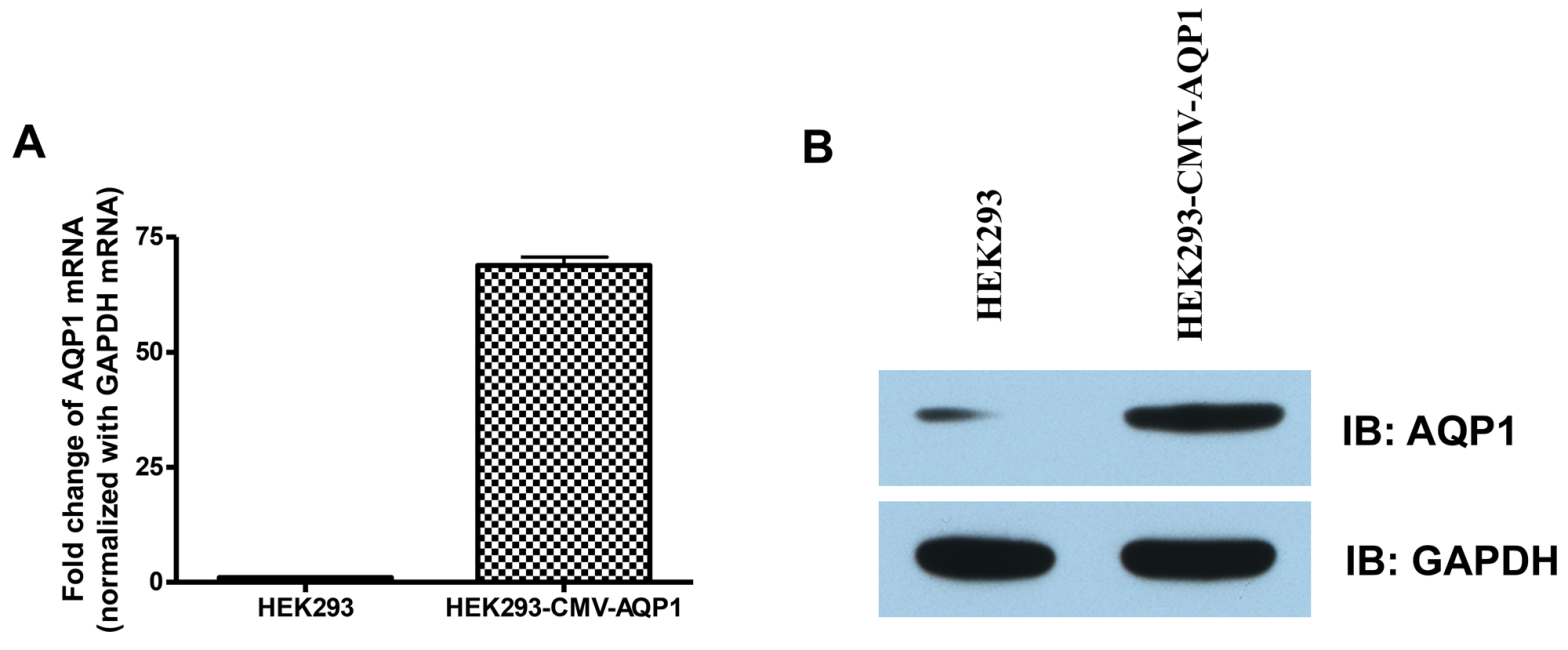
Detection of AQP1 expression in the stable HEK 293 cells. HEK293 and HEK293-CMV-AQP1 cells were collected The AQP1 **(A)** mRNAtranscription levels were quantified using RT-QPCRand **(B)** protein levels were quantifiedusing Western blotting.

We used PCR to confirm if the CMV-Neo-2A sequence was accurately integrated into the genome at the gRNA1 cleavage site. The designed specific primers (genome- and HR-specific primers) were used for amplification (Figure 4A). We found the following paired primers: 1) HR-Forward (HR-F, genome) + CMV-R (integration template) and 2) HR-Reverse (HR-R, genome) + Neo-F (integration template) amplified the expected fragment sizes (Figure 4B). To our surprise, the results alsorevealthat thepotential non-integrated, genome-specific fragment was also amplified (Figure 5A, bottom, HR-F and HR-R primer amplification). These findingssuggest that the homology directed repair was achieved only within one chromosome. Additionally, we used amplified fragment sequencing to further confirm the template integration (Figure 4C).

### CMV promoter integration increases AQP1 gene transcription and protein levels

**Figure 6:**
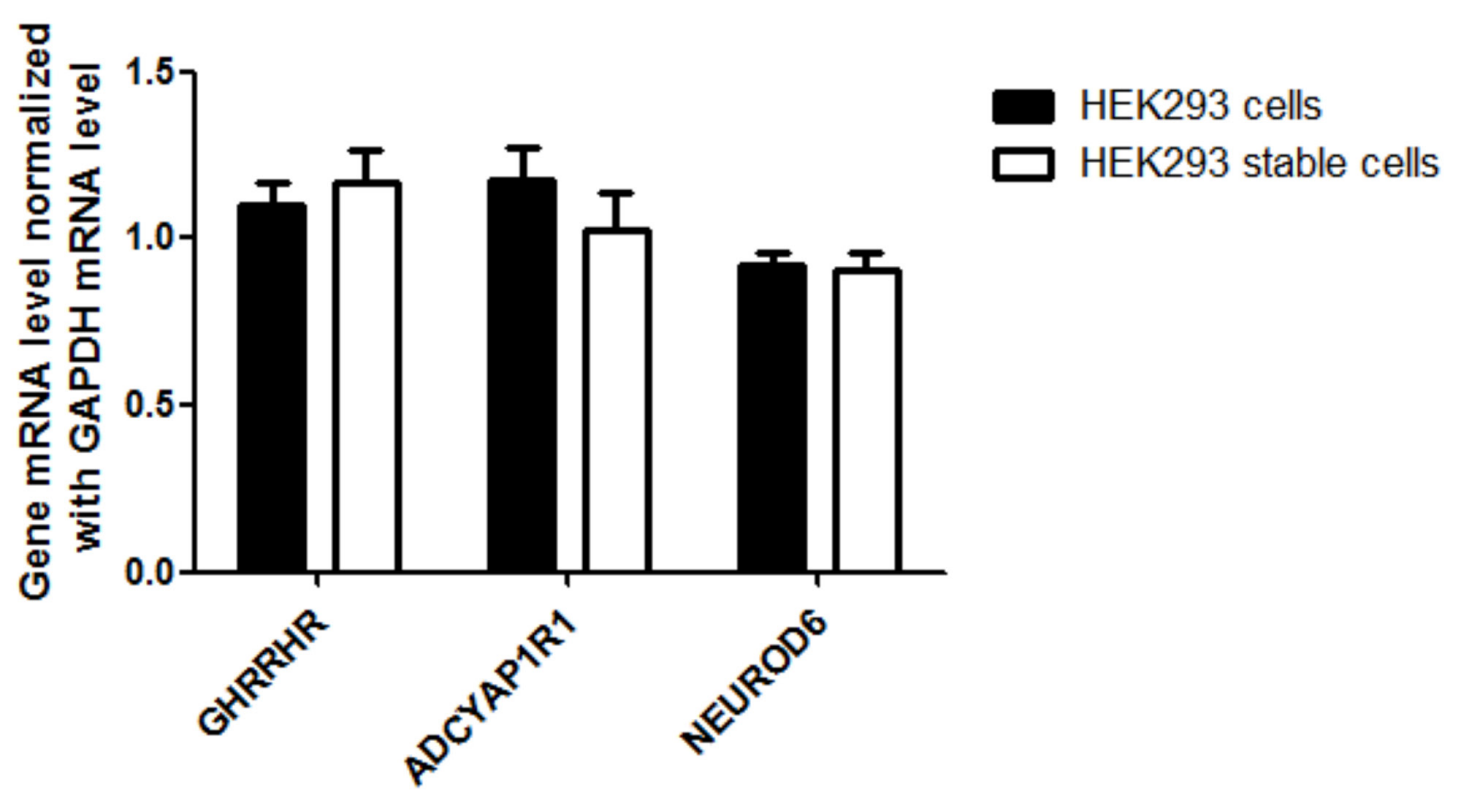
Detection of AQP1 downstream gene (GHRHR, ADCYAP1R1 and NEUROD6) expression in stable HEK 293 cells HEK293 and HEK293-CMV-AQP1 cells were collected and the mRNA levels of the genes were analyzed. The relative level of the three genes in each sample was normalized to GAPDH expression (control = 1). The experiment was run in triplicate.

We verified the integration of CMV and our next step was to determine if the stable cell line can over-express AQP1 using RT-qPCR and Western blotting. We observed a 70 fold increased transcription level and increased protein expression in the HEK293 stable cell line compared to non-integrated HEK293 control cells (Figure 5A, 5B). To confirm the transcription specificity of the CMV promoter, we measured the mRNA-level of AQP1 neighboring downstream genes (GHRHR, ADCYAP1R1 and NEUROD6) and found no significantly increased transcription levels (p>0.05, Figure 6).

### CRISPR-Cas9 system identified gRNA1 with highest cleavage efficiency in MDCK cells

**Figure 7:**
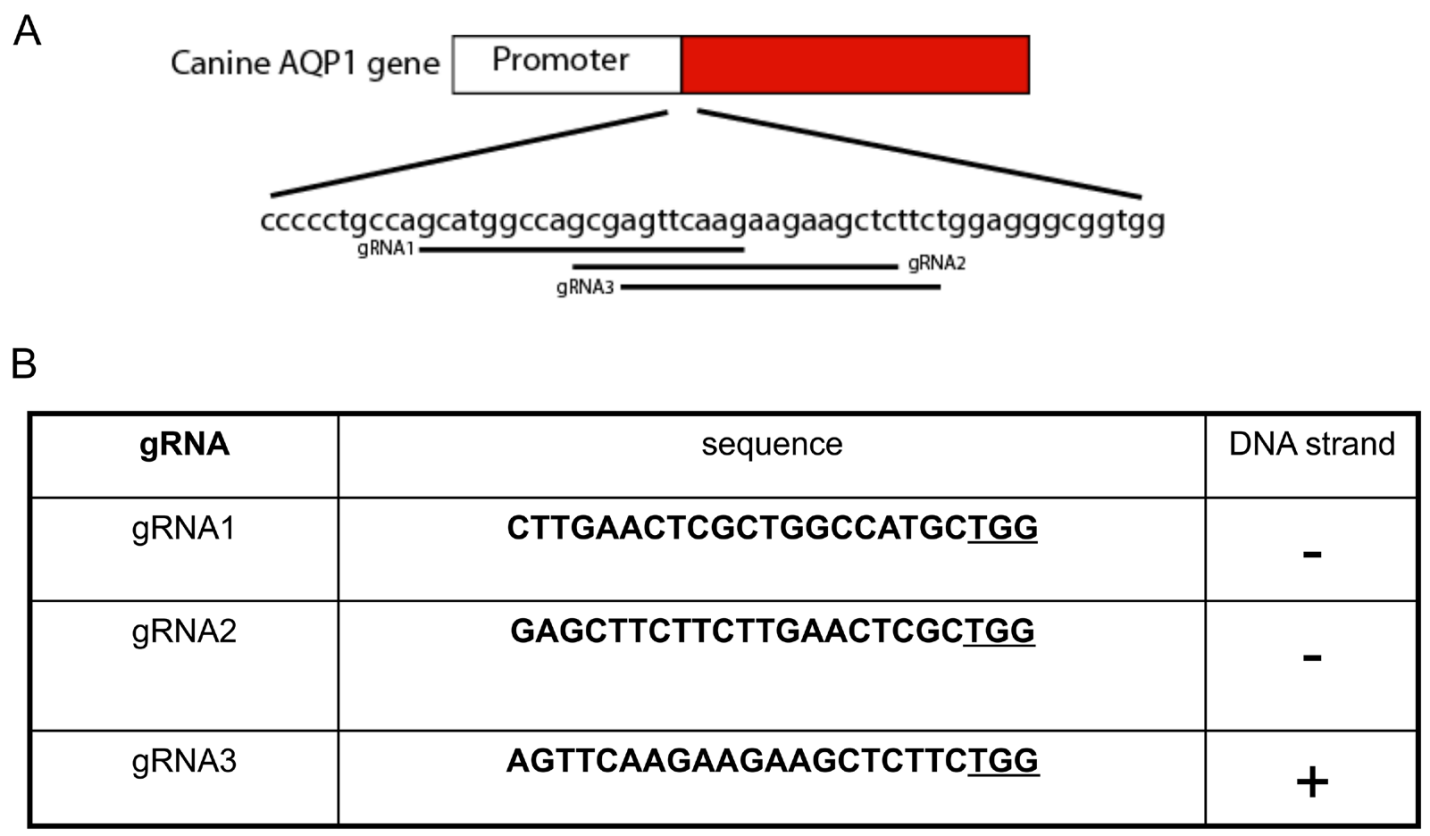
Guided RNA design for the canine AQP1 **(A)** The location of three gRNAs of canine AQP1 geneisindicated and the start codon ATG is highlighted in red. **(B)** The sequence of gRNAs and the chromosome targeting orientation along with the PAM sequence (underlined).

**Figure 8:**
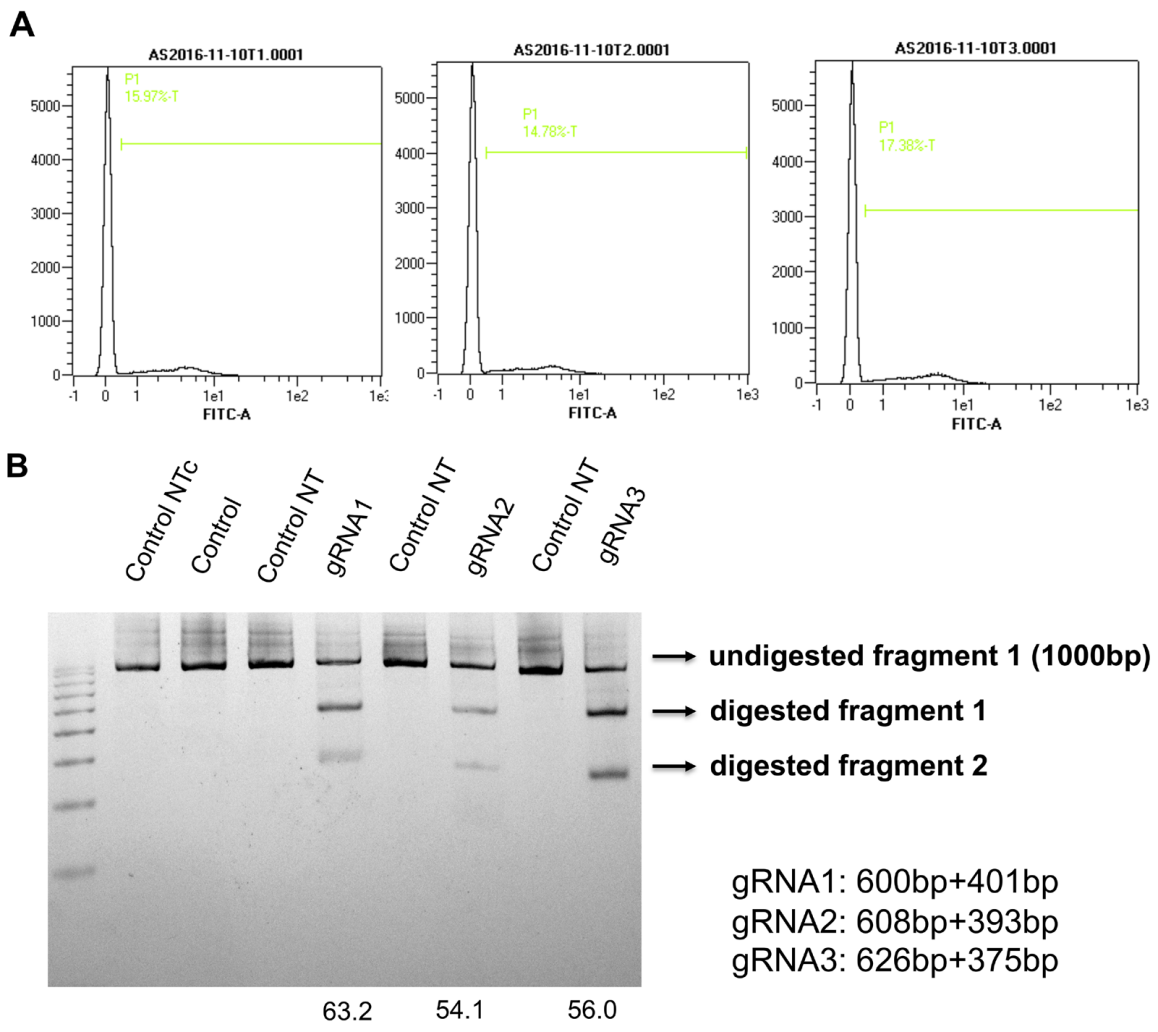
Assessing the editing efficiency of the canine AQP1 gRNAs **(A)** PX458 vectors containing canine AQP1 gRNAs weretransfected into MDCK cells and GFP positive cells were sorted by FACS. **(B)** The sorted cells and non-transfected cells (negative control) were collected, genomes DNA were isolated. The fragments crossing separategRNA sequenceswere amplified, and further digested with T7 endonuclease. Note the cleavage efficiencies were calculated and shown at the bottom of their respective gRNA-lane. The location and size of potential cleavage fragments are indicated using an arrow to the right of image.

Using the same methodsdescribed previously, three gRNAs were selected neighboring theTSS of canine AQP1 (Figure 7). After the GFP cell sort and T7Endonuclease I assay, we found that gRNA1had thehighest cleavage efficiency (gRNA1, 63.2%; gRNA2, 54/1%; gRNA3,56%)(Figure 8).

**Figure 9:**
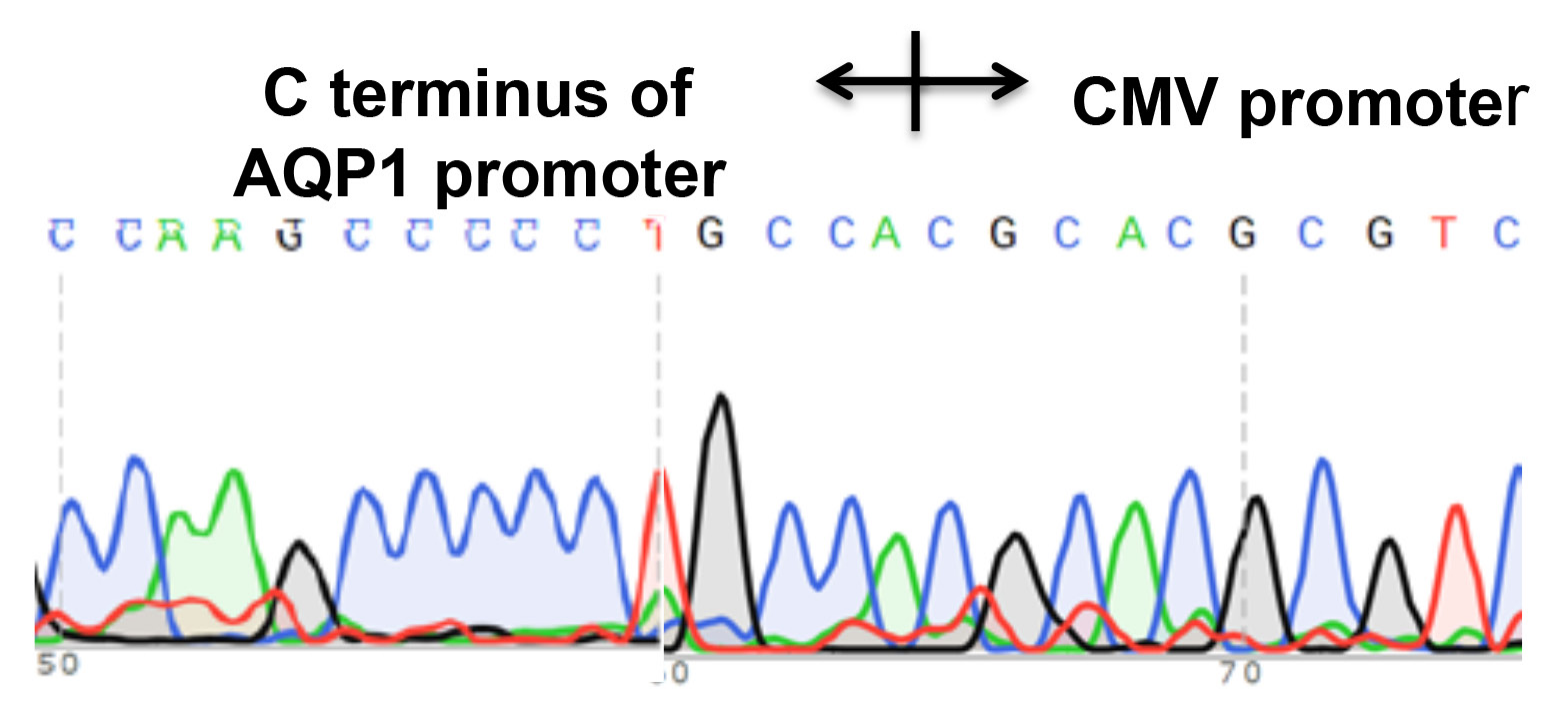
A CMV-Neo-2A fragment was accurately inserted by homologous recombination into the AQP1 genome locus The integrated stable cell line was established after PX458-gRNA1 and CMV-Neo-2A con-transfected into MDCK cells. The fragment across the gRNA1 genome sequence was amplified (primers sequence in Supplementary Figure 1). The fragment amplified was isolated by gel extraction and sequenced to confirm the CMV promoter integrated appropriately.

We integrated the CMV-Neo-2A into AQP1 gene (Supplementary Figure 3) and the stable MDCK cell line was established through theG418 antibioticselection. The primers HR-F (genome) and HR-R (integrated fragment) were used to amplify the fragment across the junction of the N-terminal of integration (Figure 9). Increased canine AQP1 transcription level was observed in the stable cells (data not shown). We didnot observe a significant difference in cell viability or growth rate between the stable integrated MDCK cells compared with normal MDCK cells after culturing for 2 months which included 30 passages (Supplementary Figures 5 and 6).

### Increased expression of canine AQP1 in MDCK cells results in increased transcellular fluid flux

We designed a functional water permeability test using the stable MDCK cell line, which has 3 distinct advantages: 1) tight junction formation in culture, 2) the entire canine genomic sequence is known, and 3) similar to humans, the canine AQP1 has also been confirmed as a water channel protein. We hypothesized that over-expression of AQP1 in a monolayer of the stable MDCK cell line would allow increased fluid flux compared to a normal MDCK cell monolayer thus providing a potential path forward in salivary gland restoration.

**Figure 10:**
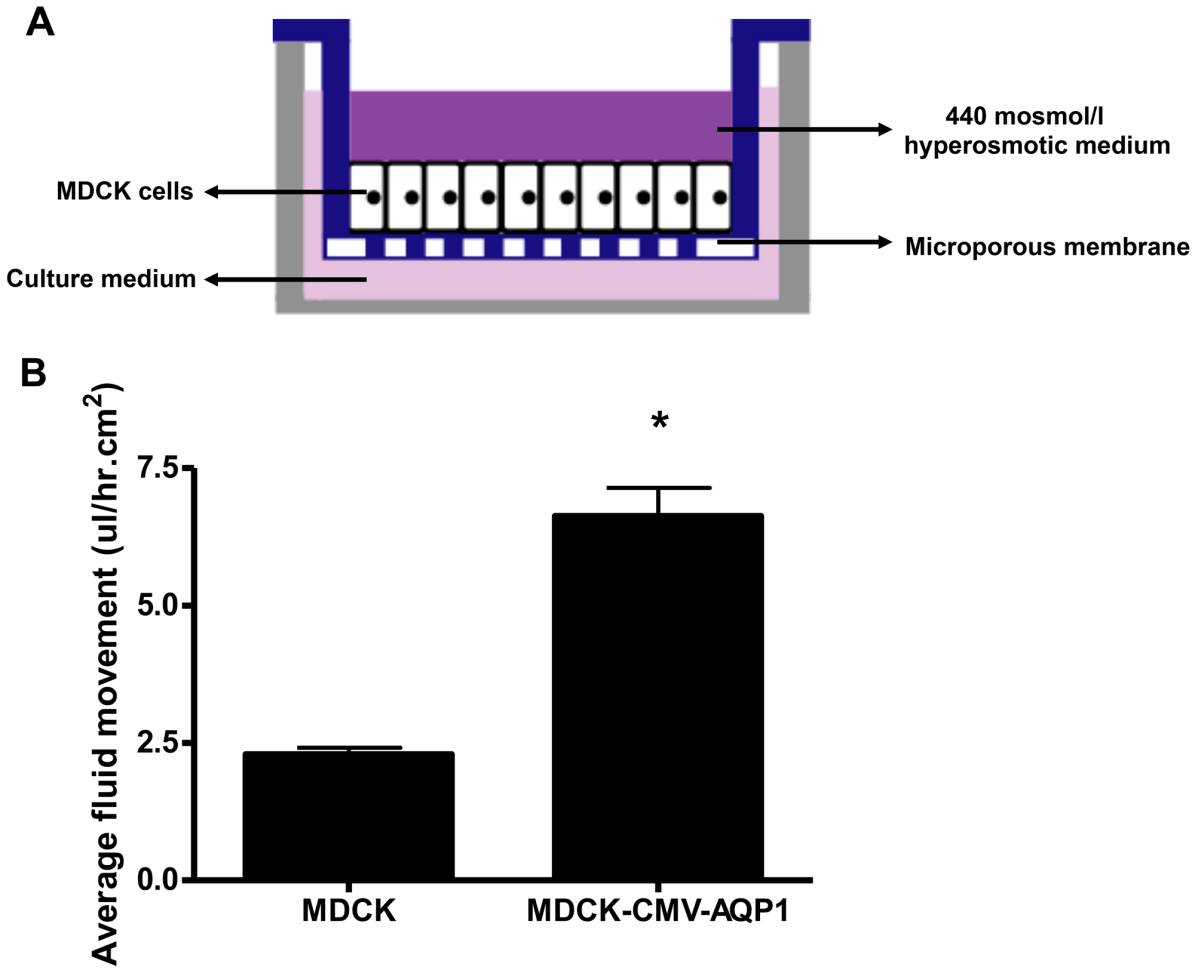
Functional assay of MDCK stable cell line: Fluid movement measurement **(A)** Schematic of the transwell culture system utilized to assess transcellular fluid flux across a confluent MDCK monolayer. The apical chamber of the well is filled with 440mOsmol/L hyperosmotic sucrose/DMEM media and the basal chamber isfilled with standard DMEM media. **(B)** The integrated stable MDCK cells had significantly greater transcellular flux of media when compared to the control MDCK cells over a 48 hour period (^*^p<0.05). Average +/− SEM.

We used a transcellular fluid flux assay to measure the functionality of our stable MDCK cell line. Using a transwell plate and hyperosmotic medium (Figure 10A) we found that MDCK cells expressing increased AQP1 also experienced increased fluid flux ((average+/-SEM) 6.4+/- 0.5ul/hr.cm^2^) when compared to normal MDCK cells (2.29+/- 0.1 ul/hr.cm^2^)(Figure 10B). These experiments demonstrate the enhanced physiological functionality of cells expressing increased AQP1 results in increased fluid flux related to permeability.

## DISCUSSION

Previous research has demonstrated that AQP1 may be anideal therapeutic target for restoring salivary gland function in cancer patients receiving ionizing radiation treatment [18]. In the present work, we expanded on this concept by using a CMV promotor to successfully increase AQP1 transcription and protein levels in both the HEK293 and MDCK stable cell lines. We also demonstrated the functional effect of increased AQP1 expression within the MDCK stable cell line resulting in increased permeability.

Many researchers have attempteddifferent method to induce over-expression of AQP1 for the treatment of salivary gland dysfunction, but each method has clinical translation limitations. Delporte, et al, used an irradiated rat salivary gland model to show efficacy and safety of using adenovirus as a delivery vector for AQP1 [11]; however, Phase II clinical trials using this method have beenwithdrawn due to a short-termeffect duration and the potential for the adenovirustoinitiate an immune response against the infected salivary gland cells (through personal talk). Our previousfindings show that we can increase the AQP1 gene expression by changing the AQP1 endogenous promoter methylation profile using thesynergistic activation mediator (SAM) CRISPR activation system; however, this system is also limited by short-term effect duration and the potential for re-demethylation of the AQP1 promoter [19]. In this study, we expanded on these ideas and were successful in increasing endogenous AQP1 expression levels, which may overcome the short-term effect duration. Compared with other existing activation systems, our endogenous promotor method allowed us to bypass epigenetic techniques allowing for strong promoter integration. This achievement alone has potentially far-reaching significance in that we can precisely control specific gene expression with various chemically- or physically-regulated promotors. Ourapproach has the ability to process alternative splicing, thus translating multiple transcripts havingvery different roles.

The selection of an appropriate promoter and the efficiency of homology directed repair must be considered. First, an appropriate promoter should be carefully chosen to meet the clinical needs. In this study, we selected CMV because it is a strong promoter and would lend to our initial, proof-of-principle experiments. Ultimately, however, promoters should be chosen based on:1) promoter methylation, 2) tissue specificity, 3) method of promoter activation (e.g. biochemical, radio waves, light), and 4) the promoter strength. Second, although the CRISPR-Cas9 system is a powerful tool to generate both knock-out celllines andanimal models, the low efficiency of homology directed repair is still a major obstacle for its further application. Yabas, et al, and Yang, et al, have shown that homologous directed repair (HDR) efficiencies less than 10% modify alleles with the majority of DSBs being repaired by NHEJ [21, 22]. We also were not able to observe an increase in AQP1 transcription and translation for transient transfection, which compelled us to create stable cell lines.

Much efforthas been made to achieve higher efficiency with different methods including: 1) synchronizing the target cells in the G2/M cycle, or 2) chemically or genetically inhibiting the NHEJ involved protein to prevent HDR repair [21, 22]. Arras, et al, recently devised a new homology-independent targeted integration (HITI) to induce robust DNA integration with high efficiency [23, 24]. Our endogenous promotor replacement method will be more efficacious with the development of these new techniques to increase HDR efficiency using CRISPR-Cas9.

In addition to our target application of treating radiation-induced salivary gland dysfunction, the methods presented provide an efficient approach to treat other human diseases or side-effects of treatments. There is potential to treat hypoglycemia by endogenously inserting a glucose inducible promoter upstream of the insulin gene [20]. Other diseases such as venous thrombosis caused by protein C promoter mutation may be corrected by replacement of new or normal promoter [25]. Also β-thalassemia caused by promoter mutation of β-globing gene also may be corrected by this promoter replacement method. [26]

Limitations exist with all scientific experimentation and we acknowledge some of those limitations, which include concern for off-target (OT) effects. Despite a reasonable number of studies, there is still no clear consensus on safety of using the CRISPR-Cas9 system. Research teams need to examine their specific targets on a case-by-case basis. For this current research, we tested two type of potential off-target effects 1) CRISCR-Cas9 cleavage off-target effect, which could bind and cut DNA sequence with imperfectly designed gRNA AQP1 promoter and 2) the possibility of neighboring downstream gene activation. In our investigation, among the ten highest potential OT sequences, no obvious cleavage bands were detected. The selection of potential OT sequence is based on a computational tool, which may not be inclusive of all off-target sequences. The most comprehensive genome wide sequencing (GWS) should be used to assess all OT sequences before use in clinical trial. Additionally, the insertion of a chosen promoter could potentially drive the expression of both target genes and other neighboringgenes. In this study, the distance between the AQP1 gene and the next neighboring downstream gene was minimal (52kb) and unlikely to be affected by the CMV promotor. However, great care should be taken to evaluate these types of side-effect andeach should be studied on a case-by-casebasis promptedbythe distance between the inserted promoter and the neighboring genes.

In summary, we provide a new gene therapy approach utilizing an endogenous promoter toinduce increased AQP1 transcription and translation. We also revealed that this increased expression ofAQP1 was functionally relevant by improving cell junction permeability. The safety and feasibility of replacingendogenous AQP1 promoter with CMV was demonstrated for the first time. This promoter integration strategy may offer an alternative and superior method for future therapies to not only alleviate side-effects of xerostemia, but also as a valid therapy for other genetic diseases.

## MATERIALS AND METHODS

### Design of three single guide RNA(sgRNA) sequences and screening of potential off-target sequences

The gene and transcript sequence for human and canine aquaporin 1 were downloaded from NCBI website and threeguided RNAs within the vicinity of the transcription start site (TSS = ATG sequence) were designed using the “CRISPR Design” software (http://crispr.mit.edu). (Figure 1) The selected gRNA sequences are listed in Table 1.

Potential off-target sequenceswerescreened using the same software. In general the off-target scores are computed by taking into account a combination of: 1) total number of genome mismatches, 2) mismatch within the absolute position, and 3) mean pairwise distance between mismatches [27]. The top ten sequences were analyzed with surveyor assay (Catalog: IC001, Genocopia TM, Rockville, MD). The selected off-target sequencesarelisted in Table 1.

### Vector construction

The plasmids used to deliver the CRISPR-Cas9 system PX458 (Addgene, depositedby Feng Zhang, PhD Massachusetts Institute of Technology, Department of Biological Engineering). The genomes targeting the oligonucleotides weresynthesized byIntegrated DNA Technology (Redwood city, CA). Each pair of oligonucleotides targeting various loci were annealed and sub-cloned within thePX458 backbone using an endonuclease restriction enzyme (BbsI, NEB, Boston, Massachusetts).

For the homologous recombinant plasmid (HR), the cytomegalo virus (CMV) promoter, Neomycin resistance gene and 2A sequence wereamplified and sub-cloned withinthe pCMV-MCS vector (Agilent Technologies, city, state). The 100bp left- and right-arm (LA and RA, respectively) fragments of the HR were amplified from genome DNA and inserted upstream of the CMV promoter (LA) and downstream of the 2A sequence (RA). The larger fragment containing LA-CMV-Neo-2A-RA was amplified and purified as a recombinanttemplate. More detailedinformation of the primers and sequences are listed in Supplementary Figure 1.

### Parallel transient transfection (HEK293) and nucleofection experiment (MDCK)

Human embryonic kidney (HEK293) cells and Madin-Darby Canine Kidney Epithelial (MDCK) cells (ATCC, Manassas, Virginia) were culturedin DMEM high glucose medium (ATCC) and MEM medium (Gibco, Shelton, Connecticut) per the supplier’s instructions.

For HEK293 cell transfection, 1X10^6^ cells were platedintoa singlewell of a 6-well plate 24 hours prior to transfection. The plated HEK293 cells were then transfected in triplicate with PX458plasmids containing: 1) the gRNA sequence targeting human AQP1 gene or 2) the gRNA sequence targeting human AQP1 gene with the homologous recombinant (HR) template fragment. The DNA-complex was created by combining 20ulPolyethylenimine (PEIPolyscienceInc, Warrington, PA) and 5ug of plasmids, in 200ul DMEM (nofetal bovine serum (FBS)) and antibiotic (10,000U/ml penicillin and streptomycin, Sigma-Aldrich, St.Louis, MO) for 10 minutes. The DNA-complex solution was pipetted into the single well containingthe cultured HEK293 cells.

For MDCK cells, nucleofection was used to deliver PX458 plasmids: 1) containing gRNA sequences targeting Canine AQP1 gene in MDCK cells or 2) together with homologous recombinant template fragment. Briefly, 5ug plasmids and 1x10^5^ MDCK cellswere mixed with 100ul Gene Pulser^@^ electroporation buffer (Bio-Rad). The nucleofection was completed with anelectoporator (Bio-Rad Laboratories, Hercules, CA) with the following settings: 150voltage, 10.0 ms pulse length, and 2mm cuvette. After nucleofection, the cells were platedin a single well of a 6-well plate using pre-warmed MEM.

After 48 hours, flow cytometry was used on both HEK293 and MDCK cultures to sort the transfected cells using GFP as a selection marker.

### Transfected cell (GFP positive) sorting using flow cytometer

Transfected cell (GFP positive) sorting was performed on the BD FACSCalibur flow cytometer (BD Biosciences, San Jose, CA) and analyzed with the Cell Quest Pro software (BD Biosciences, San Jose, CA). After 48 hours, single cell suspensions of the post-vector transfection of HEK293 or MDCK cells were prepared. A small sample of the sorted GFP positive cells were analyzed using flow cytometry to determine the sorting efficiency of the FACSCalibur.

After sorting, the transfected cells (GFP positive) were harvested and total DNA was isolated to determine highest Cas9 cleavage efficiency.

### DNA isolation and T7 endonuclease I assay (PCR) to determine Cas9 cleavage efficiency

Genomic DNA of HEK293 and MDCK transfected GFP positive cells were extracted using the QIAamp^@^ DNA mini kit (Qiagen, Beverly, MA) following the manufacturer’s protocol. In brief, harvested cells were centrifuged and the cell pellets were suspended in lysis buffer containing proteinase K. The lysate wasincubatedat 56°C for 10 min. Methanol precipitation was performed and DNA was loaded ontoamembrane column. After several rounds of washing, DNA was eluted with distilled water. The concentration of isolated DNA was quantified with Nanodrop instrument (Thermo Scientific, Waltham, MA).

The genomic region (LA: AQP1-F primer, RA: AQP1-R primer) flanking the CRISPR target site for each gene was PCR amplified (Figure 4) using a hotstartTag^@^ DNA polymerase kit (Qiagen, Beverly, MA). Products were purified using a QIAquick^@^PCR purification kit (Qiagen, Beverly, MA) following the manufacturer’s protocol. Briefly, 200 ng of purified PCR products were mixed with 2 ul 10xNEB Buffer2 and ultrapure water to a final volume of 19ul. The buffer with PCR products was then subjected to a re-annealing process to enable heteroduplex formation using the following temperature and time sequence: 95°C for 5 min; 95°C to 85°C ramping at –2°C/s; 85°C to 25°C at – 0.1°C/s; and 4°C hold. After re-annealing, the products were treated with 1ul T7 endonuclease I at 37°Cfor 15 minutes and loaded ontoa 4% mini-preotean^@^ TBE precast gel (Bio-Rad Laboratories, Hercules, CA). Gels were stained with a Sterling^@^ rapid silver staining kit (National Diagnostics, Atlanta, GA) and imaged with theGel Doc gel imaging system (Bio-Rad Laboratories, Hercules, CA). Cleavage efficiency was quantified using relative bandintensities (Image J, NIH, https://imagej.nih.gov/ij/) of the undigested PCR product (a) and the cleavage products (b and c), which were then used to calculate the percent gene modification using the equation: (1 – (1 – (b + c)/(a + b + c))^1/2^)^*^100%.

### RNA isolation and T7 endonuclease I assay (PCR) tovalidate the stable cell lines

The PX458 with the highest cleavage efficiency and the HR plasmid delivered cells were transfected for 72 hours and then treated with G418 antibiotic (final concentration, 500ug/ml) for 2 weeks. Any non-transfected cells or transfectedcells without the homologous recombinants are unable to tolerate the G418 antibiotic and eliminated, thus establishing the stable cell lines (HEK293 and MDCK). After reaching confluence, the stable cell lines (GFP positive and inserted neomyocin resistance gene) were passaged once and harvested. Total RNA and protein were isolated from the stable cell lines to measure AQP1 transcription and protein levels. Additionally, the stable MDCK cells werealso used for the functional fluid fluxassay to assess permeability.

### Cell proliferation assay

The cell proliferation assay was conducted on the stable cell lines HEK293 and MDCK (G418 resistant). Briefly, 2x10^4^ cells were plated into one well of a 24 well plate. After 5 days, the cells were harvested usingTrypsin-EDTA (0.05%) (Thermo Scientific, Waltham, MA). We used a hemocytometer to quantify the final cell concentrations.

### Cell viability assay

The cell viability assay was conducted on the stable cell lines (HEK293 and MDCK) integrated with CMV and cultured for15 passages. The CMV integrated HEK293 (1x10^5^ cells) and MDCK cells (1x10^5^ cells) were plated in 24 well plates. Cell viability was assessed using theViaQuantTM viability kit (GenecopoeiaTM, Rockville, MO). Briefly, after 24 hours, calcein AM and propidium iodide (PI) were mixed in PBS to reach final concentrations of 2uM and 4uM, respectively. The two probe mixer was added to the cells for 30 minutes. The polyanionic dye calcein is well retained within live cells, producing an intense uniform greenfluorescence in live cells (Ex/Em ∼495 nm/∼520 nm), while propidium iodide (PI) enters cells with damagedmembranes and undergoes a 30-fold enhancement of fluorescence upon binding to nucleic acids, thereby producing a bright red fluorescence in dead cells (Ex/Em ∼528 nm/∼617 nm). In order to quantify cell viability, we randomly selected 1000 cells using a fluorescence imaging system (EVOS FL Auto, Waltham, MA Thermo Fisher Scientific) and counted the number of dead (RFP) versus live cells (GFP).

### AQP1 transcription level assays: total RNA isolation and quantitative real-time PCR

The stable cellswereharvested and total RNA was isolated using the RNeasy mini kit (Qiagen, Beverly, MA). Using oligo dT primers, 1 μg total RNA was used to generate first-strandcomplementary DNA (cDNA)(First Strand cDNA Synthesis kit; Roche Life Science). Quantitative PCR (qPCR) was carried out usinga LightCycler 480 system (Roche Life Sciences). The AQP1 primer sequences used were as following: forward, 5’-CTCTCAGGCATCACCTCCTC-3’; reverse: 5’-GGAGGGTCCCGATGATGATCT-3’. Quantitative gene expression data of glyceraldehyde 3-phosphate dehydrogenase (GAPDH) was used as a reference for AQP1 expression. The GAPDH primer sequences were as follows: forward, 5’-CATGGGTGTGAACCATGAGAA-3’; reverse: 5’-GGTCATGAGTCCTTCCACGAT-3’. Quantitative PCR amplification temperature and time sequence : 95°C for 5 min followed by 45 cycles at 95°C for 10 s, 60°C for 10 s, and 72°C for 10 s. The relative mRNA level of AQP1 in each sample was normalized toGAPDH expression, and relative mRNA levels were determined by setting the control samples as 1.

### AQP1 protein level assay: western immunoblotting

Total protein levels were detected by Western Immunoblotting. Cells were lysed inRIPA buffer (150mM sodium chloride, 1% Triton X-100, 0.1%SDS, 0.5% sodium deoxycholate, 50mM Tris, pH8.0). Whole cell lysates were subjected to protein quantification using the Bradford method. Equal amounts of protein were resolved by SDS-PAGE, transferred to nitrocellulose membranes, and immunoblotted with mouse anti-human AQP1 antibody (1:500 dilution; Abcam, Cambridge MA) and GAPDH (1:5,000 dilution; Millipore, Temecula, CA). The nitrocellulose membraneswere incubated with mouse horse radish peroxidase (HRP) conjugated secondary antibody (1:10,000, Jackson ImmunoResearch, West Grove, PA) and developed with enzyme-linked chemiluminescence.

### Functional assay of MDCK stable cell line: transcellular fluid flux

A transcellular fluid flux assay was used to evaluate the canineAQP1(cAQP1) channel function in the G418 selected MDCK stable cell line (Control: GFP positive; Experimental: GFP positive and inserted neomyocin resistance gene). A difference in fluid flux between the experimental and control cells would suggest that any altered AQP1 expression affected the permeability functionality of the cells. The stable cell lines were platedonto a collagen-coated polycarbonate filter within a transwell system (Corning, Tewksbury, MA, USA) as shown in Figure 5A. Upon reaching confluence, the media within the apical (upper) chamber was replaced with 1.5 ml hyperosmotic sucrose/MEM (440 mOsmol/L) and the mediain the basal (lower) chamber was replaced with 2.6ml MEM. After 48hours, the fluid volume in the individual apical chambers was measured by pipette and the net transcellular fluid fluxwas calculated (ul/hr.cm^2^).

### Statistical analysis

The data was analyzed using a 1-way analysis of variance (ANOVA) and *post hoc* Scheffe test. A P value of <0.05 was considered significant.

## SUPPLEMENTARY MATERIALS FIGURES



## References

[1] Stepnick D, Gilpin D (2010). Head and neck cancer: an overview. Semin Plast Surg.

[2] Grundmann O, Mitchell GC, Limesand KH (2009). Sensitivity of salivary glands to radiation: from animal models to therapies. J Dent Res.

[3] Acauan MD, Figueiredo MA, Cherubini K, Gomes AP, Salum FG (2015). Radiotherapy-induced salivary dysfunction: structural changes, pathogenetic mechanisms and therapies. Arch Oral Biol.

[4] Vissink A, van Luijk P, Langendijk JA, Coppes RP (2015). Current ideas to reduce or salvage radiation damage to salivary glands. Oral Dis.

[5] Nam K, Wang CS, Maruyama CL, Lei P, Andreadis ST, Baker OJ (2017). L1peptide-conjugated fibrin hydrogels promote salivary gland regeneration. J Dent Res.

[6] Ogawa M, Oshima M, Imamura A, Sekine Y, Ishida K, Yamashita K, Nakajima K, Hirayama M, Tachikawa T, Tsuji T (2013). Functional salivary gland regeneration by transplantation of a bioengineered organ germ. Nat Commun.

[7] Teos LY, Zheng CY, Liu X, Swaim WD, Goldsmith CM, Cotrim AP, Baum BJ, Ambudkar IS (2016). Adenovirus mediated hAQP1 expression in irradiated mouse salivary glands causes recovery of saliva secretion by enhancing acinar cell volume decrease. Gene Ther.

[8] Braddon VR, Chiorini JA, Wang S, Kotin RM, Baum BJ (1998). Adenoassociated virus-mediated transfer of a functional water channel into salivary epithelial cells *in vitro* and *in vivo*. Hum Gene Ther.

[9] Wang Z, Zourelias L, Wu C, Edwards PC, Trombetta M, Passineau MJ (2015). Ultrosound-assisted nonviral gene transfer of AQP1 to the irradiated minipig parotid gland restores fluid secretion. Gene Ther.

[10] Wang Z, Pradhan-Bhatt S, Farach-Carson MC, Passineau MJ (2017). Artificial induction of native aquaporin-1 expression in human salivary cells. J Dent Res.

[11] Delporte C, O’Connell BC, He X, Lancaster HE, O’Connell AC, Agre P, Baum BJ (1997). Increased fluid secretion after adenoviral-mediated transfer of the aquaporin-1 cDNA to irradiated rat salivary glands. Proc Natl Acad Sci U S A.

[12] Luciano A, Marraffini, Erik J (2017). Sontheimer. CRISPR interference: RNA-directed adaptive immunity in bacteria and archaea. Nat Rev Drug Discov.

[13] Fellmann C, Gowen BG, Lin PC, Doudna JA, Corn JE (2017). Cornerstones of CRISPR-Cas in drug discovery and therapy. Nat Rev Drug Discov.

[14] Harrison MM, Jenkins BV, O’Connor-Giles KM, Wildonger J (2014). A CRISPR view of development. Genes Dev.

[15] Selle K, Barrangou R (2015). Harnessing CRISPR-Cas systems for bacterial genome editing. Trends Microbiol.

[16] Li M, Zhao L, Page-McCaw PS, Chen W (2016). Zebrafish genome engineering using the CRISPR-Cas9 system. Trends Genet.

[17] Liang X, Potter J, Kumar S, Zou Y, Quintanilla R, Sridharan M, Carte J, Chen W, Roark N, Ranganathan S, Ravinder N, Chesnut JD (2015). Rapid and highly efficient mammalian cell engineering via Cas9 protein transfection. J Biotechnol.

[18] Baum BJ, Zheng C, Cotrim AP, McCullagh L, Goldsmith CM, Brahim JS, Atkinson JC, Turner RJ, Liu S, Nikolov N, Illei GG (2009). Aquaporin-1 gene transfer to correct radiation-induced salivary hypofunction. Handb Exp Pharmacol.

[19] Konermann S, Brigham MD, Trevino AE, Joung J, Abudayyeh OO, Barcena C, Hsu PD, Habib N, Gootenberg JS, Nishimasu H, Nureki O, Zhang F (2015). Genome-scale transcriptional activation by an engineered CRISPR-Cas9 complex. Nature.

[20] Xu X, Tao Y, Gao X, Zhang L, Li X, Zou W, Ruan K, Wang F, Xu GL, Hu R (2016). A CRISPR-based approach for targeted DNA demethylation. Cell Discov.

[21] Yabas M, Elliott H, Hoyne GF (2015). The role of alternative splicing in the control of immune homeostasis and cellular differentiation. Int J Mol Sci.

[22] Yang D, Scavuzzo MA, Chmielowiec J, Sharp R, Bajic A, Borowiak M (2016). Enrichment of G2/M cell cycle phase in human pluripotent stem cells enhances HDR-mediated gene repair with customizable endonucleases. Sci Rep.

[23] Arras SD, Fraser JA (2016). Chemical inhibitor of non-homologous end joining increase targeted construct integration in cryptococcusneoformans. PLoS One.

[24] Suzuki K, Tsunekawa Y, Hernandez-Benitez R, Wu J, Zhu J, Kim EJ, Hatanaka F, Yamamoto M, Araoka T, Li Z, Kurita M, Hishida T, Li M (2016). *In vivo* genome editing via CRISPR/Cas9 mediated homology-independent targeted integration. Nature.

[25] Sperk CA, Greengard JS, Griffin JH, Bertina RM, Reitsma PH (1995). Two mutations in the promoter region of the human protein C gene both cause type i protein c deficiency by disruption of two HNF-3 binding sites. J Biol Chem.

[26] Ropero P, Erquiaga S, Arrizabalaga B, Pérez G, de la Iglesia S, Torrejón MJ, Gil C, Elena C, Tenorio M, Nieto JM, de la Fuente-Gonzalo F, Villegas A, González Fernández FA, Martínez R (2017). Phenotype of mutations in the promoter region of the β-globin gene. J Clin Pathol.

[27] Cong L, Ran FA, Cox D, Lin S, Barretto R, Habib N, Hsu PD, Wu X, Jiang W, Marraffini LA, Zhang F (2013). Multiplex genome engineering using CRISPR/Cas systems science.

[28] Bannister SC, Wise TG, Cahill DM, Doran TJ (2007). Comparison of chicken 7SK and U6 RNA polymerase III promoters for short hairpin RNA expression. BMC Biotechnol.

