# Supplementary material for: CRISPR-Cas9 HDR system enhances AQP1 gene expression
Source: Oncotarget. 2017 Dec 4;8(67):111683–96. doi: 10.18632/oncotarget.22901 (PMC5762352; doi:10.18632/oncotarget.22901)
Supplement: Supplementary file 1 [file oncotarget-08-111683-s001.pdf]

CRISPR-Cas9 HDR system enhances AQP1 gene expression

SUPPLEMENTARY MATERIALS

Primers for PCR denogenous human AQP1 TSS region to detect DSB in HEK293

5'-GAGGAGGTCTGTGTGGTGTG-3'

5'-CACAGTGCCTGGCAGAAAAC-3'

Primers for PCR CMV-Neo integration from upstream in HEK293 cells

5'-GAGGAGGTCTGTGTGGTGTG-3'

5'-CCGTAAGTTATGTAACGCGG-3'

Primers for PCR CMV-Neo integration from downstream in HEK293 cells

5'-ACCTTGCTCCTGCCGAGAAAGTAT-3'

5'-CACAGTGCCTGGCAGAAAAC-3'

Primers for PCR CMV-Neo integration from upstream in MDCK cells

5'-GGCTGTGGCTCAGCTCTC-3'

5'-CGTCATTATTGACGTCAATG-3'

Primers for RT-qPCR human AQP1 gene:

5'-CTCTCAGGCATCACCTCCTC

5'-GGAGGGTCCCGATGATCT

Primers for RT-qPCR human GAPDH:

5'-CATGGGTGTGAACCATGAGAA-3'

5'-GGTCATGAGTCCTTCCACGAT-3'

Primers for RT-qPCR GHRHR gene:

5'-GGCACTCTCATCCTCTCTCAG-3'

5'-CACGTCTTCTGCGTGTTGAG-3'

Primers for RT-qPCR ADCYAP1R1:

5'-CTGACCAGGACCATCTCACC-3'

5'-AGCTGATGGGCTTCAATGAT-3'

Primers for RT-qPCR NEUROD6:

5'-TGGCATTACAACAGACTCATCA-3'

5'-TTGGAAGACCCAAAATGAAA-3'

Primers for PCR denogenous AQP1 to detect DSB in MDCK cells

5'-GGTGTGGGAGACCCAGAATG-3'

5'-CACCCACGTACCTCGTTTC-3'

Primers for RT-qPCR canine GAPDH:

5'-TGTCCCCACCCCAATGTATC-3'

5'-CTCCGATGCCTGCTTCACTACCTT-3'

Supplementary Figure 1: List of primers used in the paper.

CCCCGCCCCCGGCCCTATAAATAGGCCAGCCAGGCTGTGGCTCAGCTCTCAGAGGG
AATTGAGCACCCGGCAGCGGTCTCAGGCCAAGCCCCCTGCCA

HR Left Arm

CGCACGCGTCTAGTTATTAATAGTAATCAATTACGGGGTCATTAGTTCATAGC
CCATATATGGAGTTCGCGTTACATAACTTACGGTAAATGGCCCGCCTGGCTG
ACCGCCCAACGACCCCCGCCATTGACGTCAATAATGACGTATGTTCCCAT
GTAACGCCAATAGGGACTTTCCATTGACGTCAATGGGTGGAGTATTTACGGT
AAACTGCCCACTTGGCAGTACATCAAGTGTATCATATGCCAAGTACGCCCC
TATTGACGTCAATGACGGTAAATGGCCCGCCTGGCATTATGCCCAGTACATG
ACCTTATGGGACTTTCTACTTGGCAGTACATCTACGTATTAGTCATCGCTAT
TACCATGGTGATGCGGTTTTTGGCAGTACATCAATGGGCGTGGATAGCGGTTT
GACTCACGGGGATTTCCAAGTCTCCACCCCATGACGTCAATGGGAGTTTGT
TTGGCACCAAAATCAACGGGACTTTCCAAAATGTCGTAACAACTCCGCCCCA
TTGACGCAAATGGGCGGTAGGCGTGTACGGTGGGAGGTCTATATAAGCAGAG
CTCGTTTAGTGAACCGTCAGATCGCCTGGAGACGCCATCCACGCTGTTTTGAC
CTCCATAGAAGACACCGGGACCGATCCAGCCTCCG

CMV Promoter

ATGATTGAACAAGATGGATTGCACGCAGGTTCTCCGGCCGCTTGGGTGGAGA
GGCTATTCGGCTATGACTGGGCACAACAGACAATCGGCTGCTCTGATGCCGC
CGTGTTCCGGCTGTCAGCGCAGGGGGCGCCCGGTTCTTTTTGTCAAGACCGACC
TGTCGGTGCCCTGAATGAACTGCAGGACGAGGCAGCGCGGCTATCGTGGCT
GGCCACGACGGGCGTTCCTTGCGCAGCTGTGCTCGACGTTGTCACTGAAGCG
GGAAGGGACTGGCTGCTATTGGGCGAAGTGCCGGGGCAGGATCTCCTGTCAT
CTCACCTTGCTCCTGCCGAGAAAGTATCCATCATGGCTGATGCAATGCGGCG
GCTGCATACGCTTGATCCGGCTACCTGCCCATTCGACCACCAAGCGAAACAT
CGCATCGAGCGAGCACGTACTCGGATGGAAGCCGGTCTTGTCGATCAGGATG
ATCTGGACGAAGAGCATCAGGGGCTCGCGCCAGCCGAACTGTTCCGCCAGGCT
CAAGGCGCGCATGCCCGACGGCGAGGATCTCGTCGTGACCCATGGCGATGCC
TGCTTGCCGAATATCATGGTGGAAAATGGCCGCTTTTCTGGATTTCATCGACTG
TGGCCGGCTGGGTGTGGCGGACCGCTATCAGGACATAGCGTTGGCTACCCGT
GATATTGCTGAAGAGCTTGGCGGCGAATGGGCTGACCGCTTCCTCGTGCTTTA
CGGTATCGCCGCTCCCGATTGCGCAGCGCATCGCCTTCTATCGCCTTCTTGACG
AGTTCCTC

Neomycin cDNA

GGAAGCGGAGCTACTAACTTCAGCCTGCTGAAGCAGGCTGGCGACGTGGAGG
AGAACCCTGGACCT

P2A Sequence

ATGGCCAGCGAGTTCAAGAAGAAGCTCTTCTGGAGGGCAGTGGTGGCCGAGTTCCTGGC
CACGACCCTCTTTGTCTTCATCAGCATCGGTTCTGCCCTG

Right Arm

Supplementary Figure 2: Donor sequence for homologous recombination of HEK293 AQP1 TSS region.

CCTCCCCCGGGCCCTATAAATAGGCCAGCCCGGGCTGTGGCTCAGCTCTCGGAGGGA
GTCGAGCACCAGGCAGCGGTCTCAAGCCAAGCCCCCTGCCA

Left Arm

CGCACGCGTCTAGTTATTAATAGTAATCAATTACGGGGTCATTAGTTCATAGC
CCATATATGGAGTTCGCGTTACATAACTTACGGTAAATGGCCCGCCTGGCTG
ACCGCCCAACGACCCCCGCCATTGACGTCAATAATGACGTATGTTCCCAT
GTAACGCCAATAGGGACTTTCCATTGACGTCAATGGGTGGAGTATTTACGGT
AAACTGCCCAGTTGGCAGTACATCAAGTGTATCATATGCCAAGTACGCCCC
TATTGACGTCAATGACGGTAAATGGCCCGCCTGGCATTATGCCCAGTACATG
ACCTTATGGGACTTTTCTACTTGGCAGTACATCTACGTATTAGTCATCGCTAT
TACCATGGTGTATGCGGTTTGGCAGTACATCAATGGGCGTGGATAGCGGTTT
GACTCACGGGGATTTCCAAGTCTCCACCCCATGACGTCAATGGGAGTTTGT
TTGGCACCAAATCAACGGGACTTTCCAAATGTCGTAACAACTCCGCCCCA
TTGACGCAAATGGGCGGTAGGCGTGTACGGTGGGAGGTCTATATAAGCAGAG
CTCGTTTAGTGAACCGTCAGATCGCCTGGAGACGCCATCCACGCTGTTTGAC
CTCCATAGAAGACACGGGACCGATCCAGCCTCCG

CMV Promoter

ATGATTGAACAAGATGGATTGCACGCAGGTTCTCCGGCCGCTTGGGTGGAGA
GGCTATTCGGCTATGACTGGGCACAACAGACAATCGGCTGCTCTGATGCCGC
CGTGTTCCGGCTGTCAGCGCAGGGGCGCCCGGTTCTTTTTGTCAAGACCGACC
TGTCGGGTGCCCTGAATGAACTGCAGGACGAGGCAGCGCGGCTATCGTGGCT
GGCCACGACGGGCGTTCCTTGCGCAGCTGTGCTCGACGTTGTCACTGAAGCG
GGAAGGGACTGGCTGCTATTGGGCGAAGTGCCGGGGCAGGATCTCCTGTCAT
CTCACCTTGCTCCTGCCGAGAAAGTATCCATCATGGCTGATGCAATGCGGCG
GCTGCATACGCTTGATCCGGCTACCTGCCCATTCGACCACCAAGCGAAACAT
CGCATCGAGCGAGCACGTACTCGGATGGAAGCCGGTCTTGTCGATCAGGATG
ATCTGGACGAAGAGCATCAGGGGCTCGCGCCAGCCGAAGTTCGCCAGGCT
CAAGGCGCGCATGCCCCGACGGCGAGGATCTCGTCGTGACCCATGGCGATGCC
TGCTTGCCGAATATCATGGTGGAAAATGGCCGCTTTTCTGGATTCATCGACTG
TGGCCGGCTGGGTGTGGCGGACCGCTATCAGGACATAGCGTTGGCTACCCGT
GATATTGCTGAAGAGCTTGGCGGCGAATGGGCTGACCGCTTCCTCGTGCTTTA
CGGTATCGCCGCTCCCGATTCGCAGCGCATCGCCTTCTATCGCCTTCTTGACG
AGTTCTTC

Neomycin cDNA

GGAAGCGGAGCTACTAAGTTCAGCCTGCTGAAGCAGGCTGGCGACGTGGAGG
AGAACCCTGGACCT

P2A Sequence

AAGAAGCTCTTCTGGAGGGCGGTGGTGGCCGAGTTCCTGGCCATGATCCTCTTCGTCTT
CATCAGCATCGGTTCTGCCCTGGGCTTCAACTACCCGGTGA

Right Arm

Supplementary Figure 3: Donor sequence for homologous recombination of MDCK AQP1 TSS region.

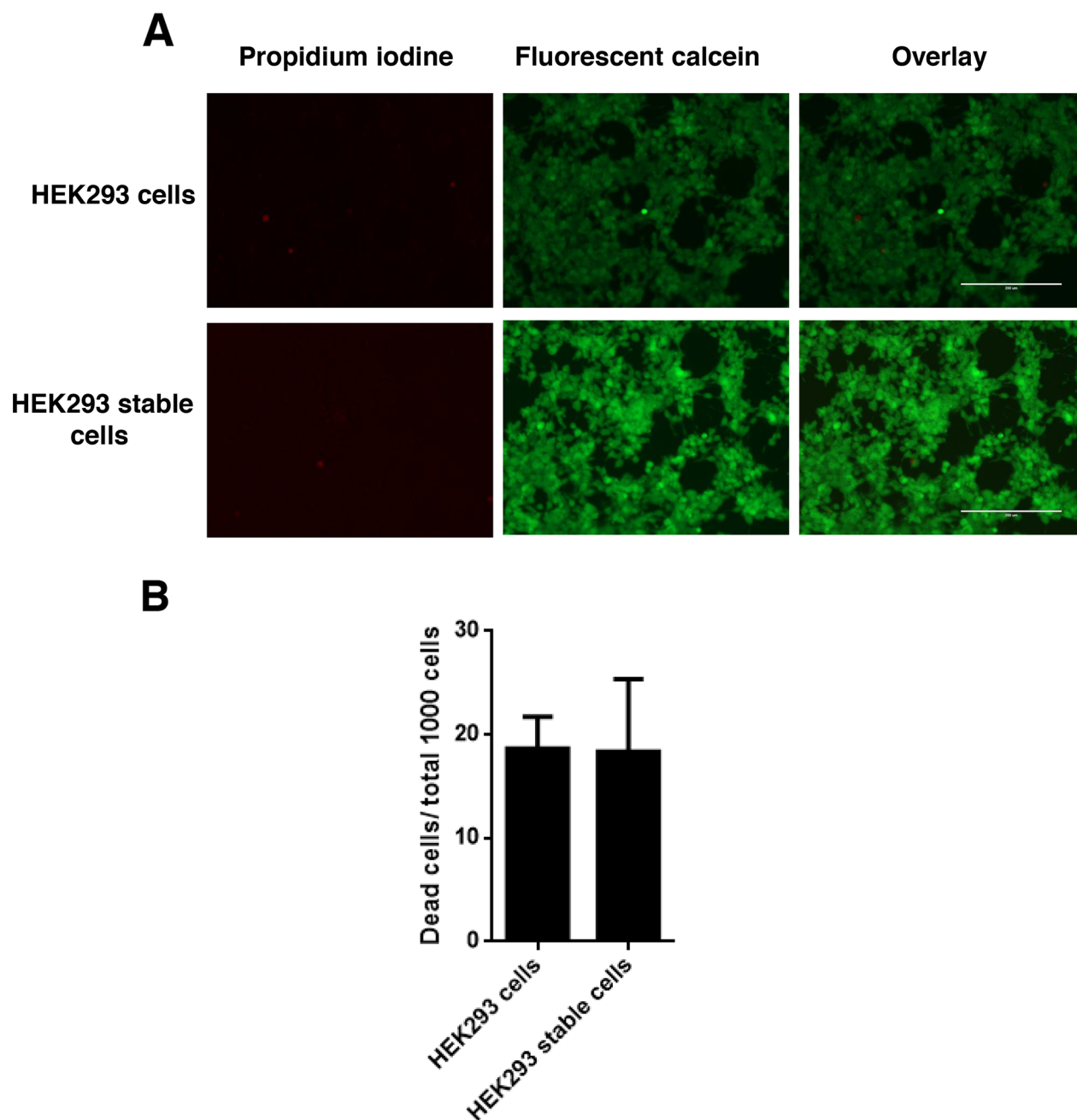

Supplementary Figure 4: The CMV integrated stable HEK293 cells have no effect on cell viability. (A) The viability of CMV integrated stable cells and normal HEK293 cells were confirmed using fluorescing Propidium iodine (RFP) and calcein (GFP) labeling to identify dead cell and live cells, respectively. (B) The dead cells per 1000 total cells were counted from Propidium iodine and calcein overlay images.

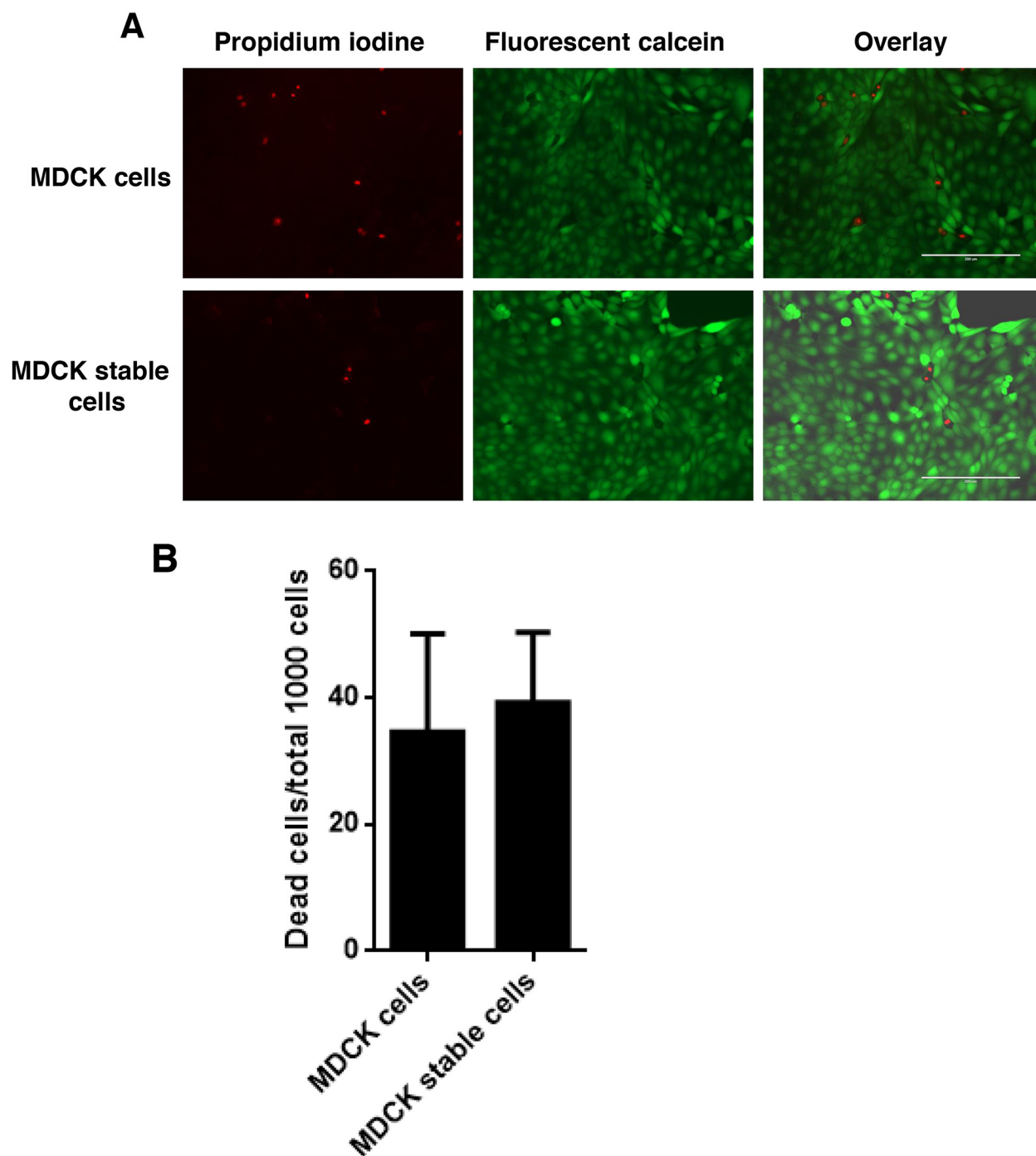

Supplementary Figure 5: CMV integrated MDCK stable cells have no effect on cell viability. (A) The viability of CMV integrated stable cells and normal MDCK cells were confirmed using fluorescing Propidium iodine (GFP) and calcein (GFP) labeling to identify dead cell and live cells, respectively. (B) The dead cells per 1000 total cells were counted from Propidium iodine and calcein overlay images.

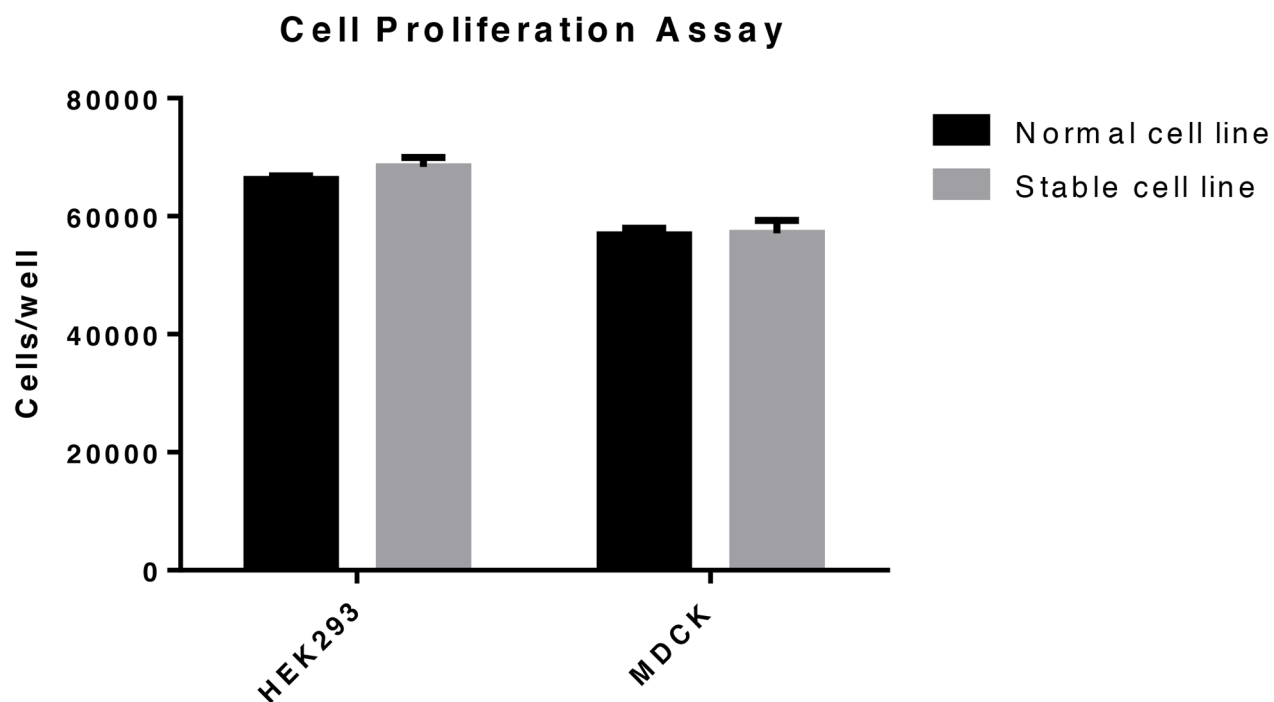

Supplementary Figure 6: CMV integrated stable HEK293 and MDCK cells have no effect on cell proliferation. The number of CMV integrated cells were counted 5 days after plating and compared to the number of normal HEK293 and MDCK cells. No significant difference was detected between stable cells and normal cells $p > 0.05$.
